# Sampling locations and processing methods shape fungi microbiome on the surface of edible and medicinal Arecae semen

**DOI:** 10.3389/fmicb.2023.1188986

**Published:** 2023-07-20

**Authors:** Guangfei Wei, Jia Xu, Zhaoyu Zhang, Guozhuang Zhang, Shilin Chen, Linlin Dong

**Affiliations:** ^1^Key Laboratory of Beijing for Identification and Safety Evaluation of Chinese Medicine, Institute of Chinese Materia Medica, China Academy of Chinese Medical Sciences, Beijing, China; ^2^Yunnan University of Traditional Chinese Medicine, Kunming, China; ^3^Shandong University of Traditional Chinese Medicine, Jinan, China; ^4^Chengdu University of Traditional Chinese Medicine, Chengdu, China

**Keywords:** edible and medicinal plant, Arecae semen, Arecae semen tostum, Arecae semen carbonisata, fungal infection

## Abstract

**Introduction:**

Arecae semen, which is derived from the dried ripe seed of *Areca catechu* L., has been commonly used as one of the major traditional Chinese medicines (TCMs). Three types of crude herbal preparations, namely, raw Arecae semen (AS), Arecae semen tostum (SAS), and Arecae semen carbonisata (FAS), are available for different clinical applications in TCMs. Although aflatoxin contamination in Arecae semen has been reported preliminarily, only a few studies have been conducted on fungal contamination.

**Methods:**

In this study, the presence of fungi on the surface of three Arecae semen (AS, SAS, and FAS) that collected from four provinces were investigated using high-throughput sequencing and internal transcribed spacer 2.

**Results:**

Results showed that the phyla Ascomycota (75.45%) and Basidiomycota (14.29%) and the genera *Wallemia* (7.56%), *Botryosphaeria* (6.91%), *Davidiella* (5.14%), and *Symbiotaphrina* (4.87%) were the dominant fungi, and they presented significant differences in four areas and three processed products (*p* < 0.05). The α-diversity and network complexity exhibited significant differences in the four sampling locations (*p* < 0.05), with higher in Yunnan (Chao 1, 213.45; Shannon, 4.61; average degree, 19.96) and Hainan (Chao 1, 198.27; Shannon, 4.21; average degree, 22.46) provinces. Significant differences were noted in the three processed samples; and SAS group had highest α-diversity (Chao 1, 167.80; Shannon, 4.54) and network complexity (average degree, 18.32).

**Conclusions:**

In conclusion, the diversity and composition of microbiome on the surface of Arecae semen were shaped by sampling location and processing methods. This work provides details on the surface microbiome of Arecae semen samples and highlights the importance of roles of origin and processing methods in microbiomes, ensuring drug efficacy and food safety.

## 1. Introduction

*Areca catechu* L., a traditional Chinese medicine, is widely distributed in tropical and subtropical provinces in southern China and other countries in South Asia and Southeast Asia (Mehrtash et al., 2017). At present, Arecae semen is commonly used as one of the major traditional Chinese medicines (TCMs) in more than 100 prescriptions (Chavan and Singhal, 2013). It has been used to treat parasites, dyspepsia, abdominal distension, abdominal pain, diarrhea, edema, and jaundice caused by a variety of chemical compounds, such as alkaloids, tannins, fats, flavonoids, carbohydrates, and crude fibers (Sun et al., 2017). The varying processing methods have led to the highly different product efficacy of Arecae semen. Three types of crude herbal preparations are available for different clinical applications in TCMs, namely, raw Arecae semen (AS), Arecae semen tostum (SAS), and Arecae semen carbonisata (FAS) (Chinese Pharmacopeia, 2020). The peel and seeds of *A. catechu* L. are not only be used as medicines, but its fruit is also widely eaten to welcome guests. Betel-quid and areca-nut chewing is widely prevalent in Chinese Taipei and Southeast Asia, and it has been the fourth most common habit after tobacco, alcohol, and caffeine-containing beverages (Lin et al., 2020). Nevertheless, the International Agency for Research on Cancer (IARC) show that this habit is carcinogenic to humans and can cause cancers of the oral cavity, pharynx, esophagus, liver, biliary tracts, and uterus (IARC, 2004).

Mycotoxins are classified as human carcinogens by the World Health Organization (WHO) Cancer Research Agency, and their toxicity is considerably higher than those of cyanide, iodide, and organic pesticides. Mycotoxins are among the most carcinogenic substances known in the world (Han et al., 2012). They are commonly found in peanuts, milk, and herbal medicines (Chen et al., 2017; Lindahl et al., 2018). The presence of mycotoxins poses a considerable immediate threat to consumer safety, because investigations have shown that even very small amounts of mycotoxins can cause a number of illnesses, including nausea, vomiting, and hepatotoxicity (Adegbeye et al., 2020; Claeys et al., 2020). Given the high temperature and humidity conditions under which Arecae semen grows, it is susceptible to the toxigenic fungi growth and mycotoxin production if improperly harvested, processed, and stored. The ultrafast liquid chromatography combined with electrospray ionization tandem mass spectrometry (UFLC-ESI-MS/MS) method was applied to detect 11 mycotoxins in 24 Arecae semen samples, and only 2 samples were positive for aflatoxin B_2_ and zearalenone (Liu et al., 2016). High-precision liquid chromatography combined with tandem mass spectrometry (HPLC-MS/MS) showed that the linear relationships between the peak area and mass concentration values of the four aflatoxins in edible areca nut were within the same range of 0.1–10.0 μg/L (Liang et al., 2018). At present, the regulation has set the maximum limits of AFB_1_ (5 μg/kg) and the sum of AFs (10 μg/kg, including AFB_1_, AFB_2_, AFG_1_, and AFG_2_) in Arecae semen in Chinese Pharmacopeia (2020).

The occurrence of fungi in herbal medicines exhibits the potential to produce mycotoxins, and it has become an interesting topic over the past decades. Aflatoxins and ochratoxin A were produced by the fungi *Aspergillus* and *Penicillium* (Niessen, 2007). One study reported that 27 of the 30 medicinal plant samples from Pakistan were contaminated with molds (Ahmad et al., 2014). The detection of toxigenic fungi in herbal medicines is important to provide early warning for mycotoxin contamination. High-throughput sequencing (HTS) is an accurate and rapid method that can provide mass data of the surface microbial composition of herbs with low abundance, such as Ziziphi Spinosae semen and Cassiae semen (Guo et al., 2018, 2020). However, only a few studies have been conducted to analyze the diversity and composition of surface fungi in Arecae semen via HTS.

In the current work, we investigated the occurrence of fungi and compared the difference of fungal diversity on the surface of samples collected from four provinces by targeting the internal transcribed spacer 2 (ITS2) through HTS. To our knowledge, this is the first paper to study the fungal microbiome on the surface of edible and medicinal Arecae semen through HTS. This study can effectively detect potential toxigenic fungi and evaluate the safety of edible and medicinal herbs. It can provide an early warning for subsequent potential mycotoxin biosynthesis.

## 2. Materials and methods

### 2.1. Sampling

A total of 36 Arecae semen samples were obtained from four provinces in China (Supplementary Table S1). These samples were divided into three groups, namely, Arecae semen (AS), Arecae semen tostum (SAS), and Arecae semen carbonisata (FAS), in accordance with different processed products. The samples were also divided into four groups, namely, Guangxi Province (GX), Hainan Province (HN), Guangdong Province (GD), and Yunnan Province (YN), on the basis of different sampling locations.

### 2.2. DNA extraction

Microorganisms on the surface of herbs were collected and DNA of microorganism was extracted according to previous report (Guo et al., 2018). Briefly, 5 g of samples and 10 mL of sterile water were transferred into a 15 mL sterile centrifuge tube and shaken for 20 min. The mixture was then filtered with sterile gauze and centrifuged at 7,830 rpm for 15 min to collect microorganisms. Total DNA was extracted using EZNA^®^ soil DNA kit (Omega Bio-tek., Inc., United States) in accordance with the manufacturer’s instructions.

### 2.3. Polymerase chain reaction amplification and HTS

The ITS2 sequence was amplified with the primer pairs ITS1FI2 (5’-GTGARTCATCGAATCTTTG-3′)/ITS2 (5’-TCCTCCGCTTATTGATATGC-3′) (Karlsson et al., 2014). PCR amplification was conducted under the following conditions: initial denaturation at 98°C for 30 s, 35 cycles of denaturation at 98°C for 10 s, annealing at 54°C for 30 s, elongation at 72°C for 45 s, and a final extension at 72°C for 10 min. The PCR products were analyzed with 2% agarose gel and purified with a DNA gel extraction kit (Axygen, United States). Purified ITS2 amplicons were sequenced with the Illumina MiSeq PE250 platform (Illumina, United States). The raw sequences were uploaded to the National Center for Biotechnology Information (NCBI) Sequence Read Archive (SRA) database (accession numbers PRJNA934825).

### 2.4. Sequence analysis

Raw FASTQ files were demultiplexed and screened for their quality with Quantitative Insights into Microbial Ecology (QIIME, version 1.8) software (Caporaso et al., 2010). Paired reads were truncated at any site to receive an average Phred quality score ≥ Q20, equivalent to a 0.01% error rate (Marasco et al., 2018). The primers were exactly matched, and two nucleotide mismatches and the reads that contained ambiguous bases were removed. A total of 2,706,500 high-quality reads (200–500 bp) were obtained from 36 samples. The obtained amplified sequence variants (ASVs) were used to analyze the diversity and taxonomic composition of Arecae semen (Izawa et al., 2020). The representative sequences of the ASVs were classified at the kingdom, phylum, class, order, family, genus, and species levels in accordance with the UNITE and INSDC databases (Kõljalg et al., 2013; Nilsson et al., 2019b).

To estimate alpha diversity, Chao 1 and Shannon indices were estimated. Beta diversity was measured using the Bray–Curtis distance matrix and then visualized via principal coordinate analysis (PCoA) and nonmetric multidimensional scaling (NMDS). Statistical differences among different groups were measured via analysis of similarities (ANOSIM). Fungal guilds were predicted using FUNGuild (Nguyen et al., 2016; Li et al., 2022a,b). Only probable and highly probable terms were used to summary the relative abundances of different guilds in each sample. Linear discriminant analysis effect size (LEfSe) with a linear discriminant analysis (LDA) score > 3.0 and *p* < 0.05 was used to discover potential discriminant microbes (Segata et al., 2011). The Circos graph was constructed with Circos software (Krzywinski et al., 2009). Co-occurrence analysis between fungal communities was conducted by Cytoscape (Faust et al., 2012). Only highly (Spearman’s |r| > 0.8) and statistically significant (*p* < 0.05) correlations were retained. The network was visualized with Gephi (Bastian et al., 2009).

## 3. Results

### 3.1. Diversity of Arecae semen surface microbiome was influenced by sampling locations and processing methods

**Figure 1:**
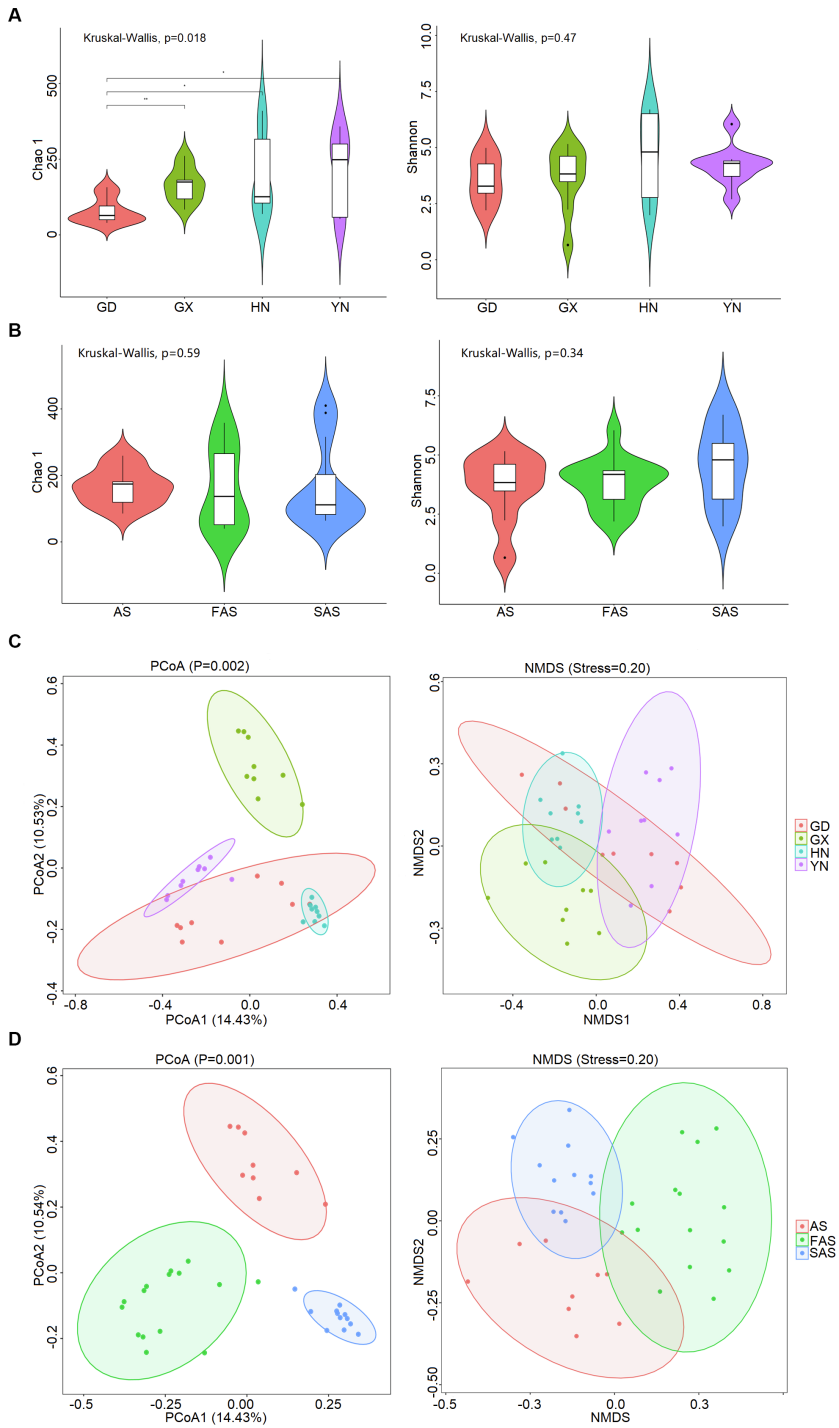
Diversity of fungal community on the surface of Arecae semen. **(A)** Chao 1 and Shannon indices based on sampling locations. **(B)** Chao 1 and Shannon indices based on processing methods. **(C)** PCoA and NMDS analysis of Bray–Curtis distance matrices based on sampling locations. **(D)** PCoA and NMDS analysis of Bray–Curtis distance matrix matrices based on processing methods.

The α-diversity of fungi on the surface of Arecae semen was strongly influenced by sampling locations (Figure 1A and Supplementary Table S2). The Chao 1 index was significantly highest in YN group (213.45 ± 41.84) and HN group (198.27 ± 44.91) than in GX group (166.11 ± 18.94) and GD group (79.08 ± 14.05) (Kruskal–Wallis, *p* < 0.05). The Shannon index was higher in HN group (4.61 ± 0.0.61) and YN group (4.21 ± 0.30) than in GX group (3.64 ± 0.48) and GD group (3.57 ± 0.34). The variation of β-diversity among samples was visualized via PCoA and NMDS (Figure 1C). The results showed that the fungal community clustered in accordance with different sampling locations (PCoA, *p* = 0.001; NMDS, stress = 20). Significant differences were found among four sampling locations (ANOSIM, R = 782).

The α-diversity was slightly affected by processing methods, but the difference was not significant (Kruskal–Wallis, *p >* 0.05; Figure 1B and Supplementary Table S3). The Chao 1 index was slightly higher in AS group (166.11 ± 18.65) and SAS group (167.80 ± 36.85) than in FAS group (160.23 ± 31.12). The Shannon index was highest in SAS group (4.54 ± 0.47) and then it incrementally decreased from FAS group (3.80 ± 0.26) to AS group (3.64 ± 0.48). In addition, the similarities of fungal communities among the three processing products were compared, and the results of PCoA and NMDS showed that the samples were clustered on the basis of processing methods (PCoA, *p* = 0.002; NMDS, stress = 0.20) and exhibited a significant difference (ANOSIM, R = 0.531; Figure 1D).

### 3.2. Composition of Arecae semen surface microbiome was influenced by sampling locations and processing methods

**Figure 2:**
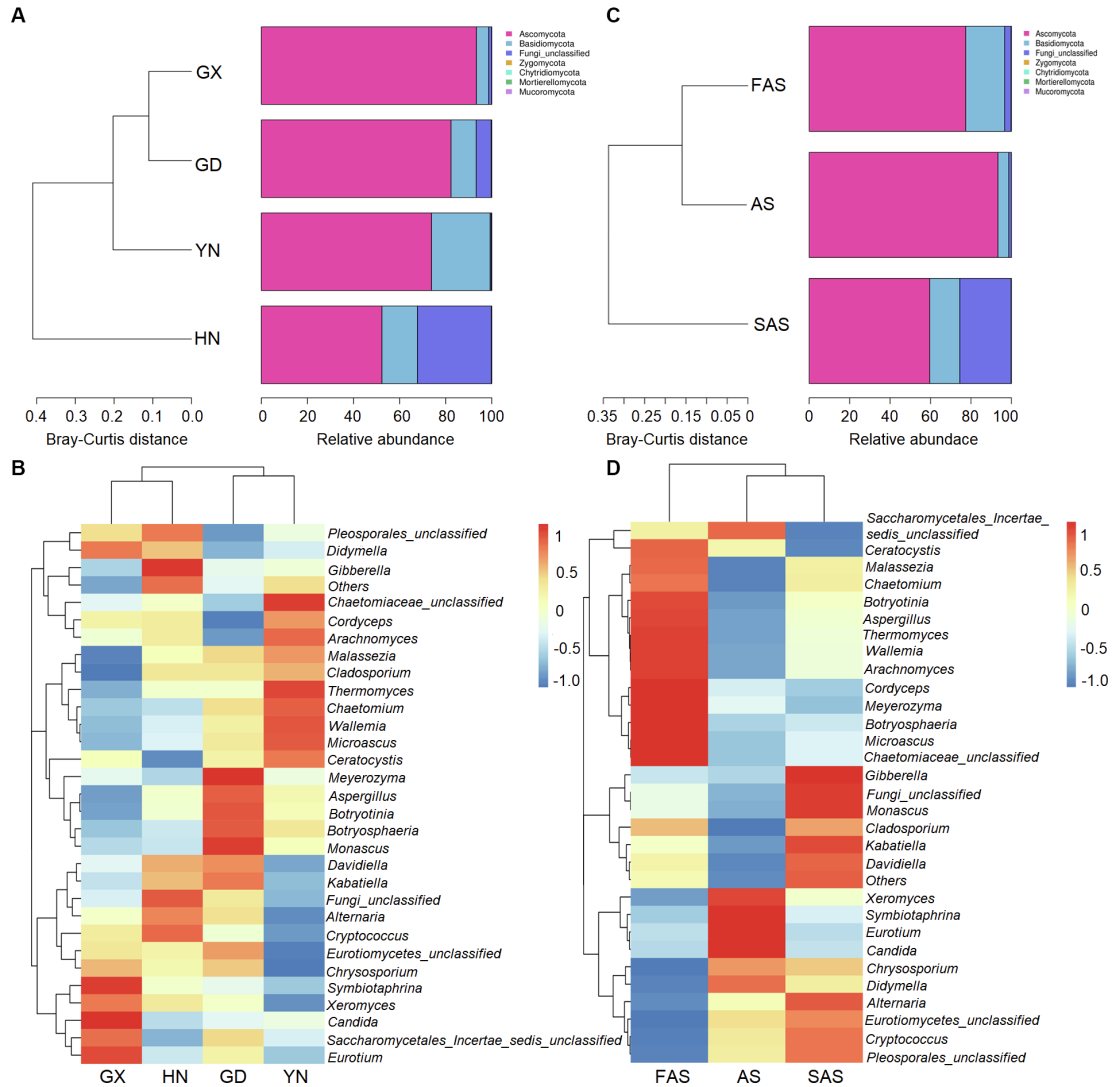
Composition of fungal community in Arecae semen samples. **(A,B)** Fungal composition at the phylum and genus level based on sampling locations. **(C,D)** Fungal composition at the phylum and genus level based on processing methods.

**Figure 3:**
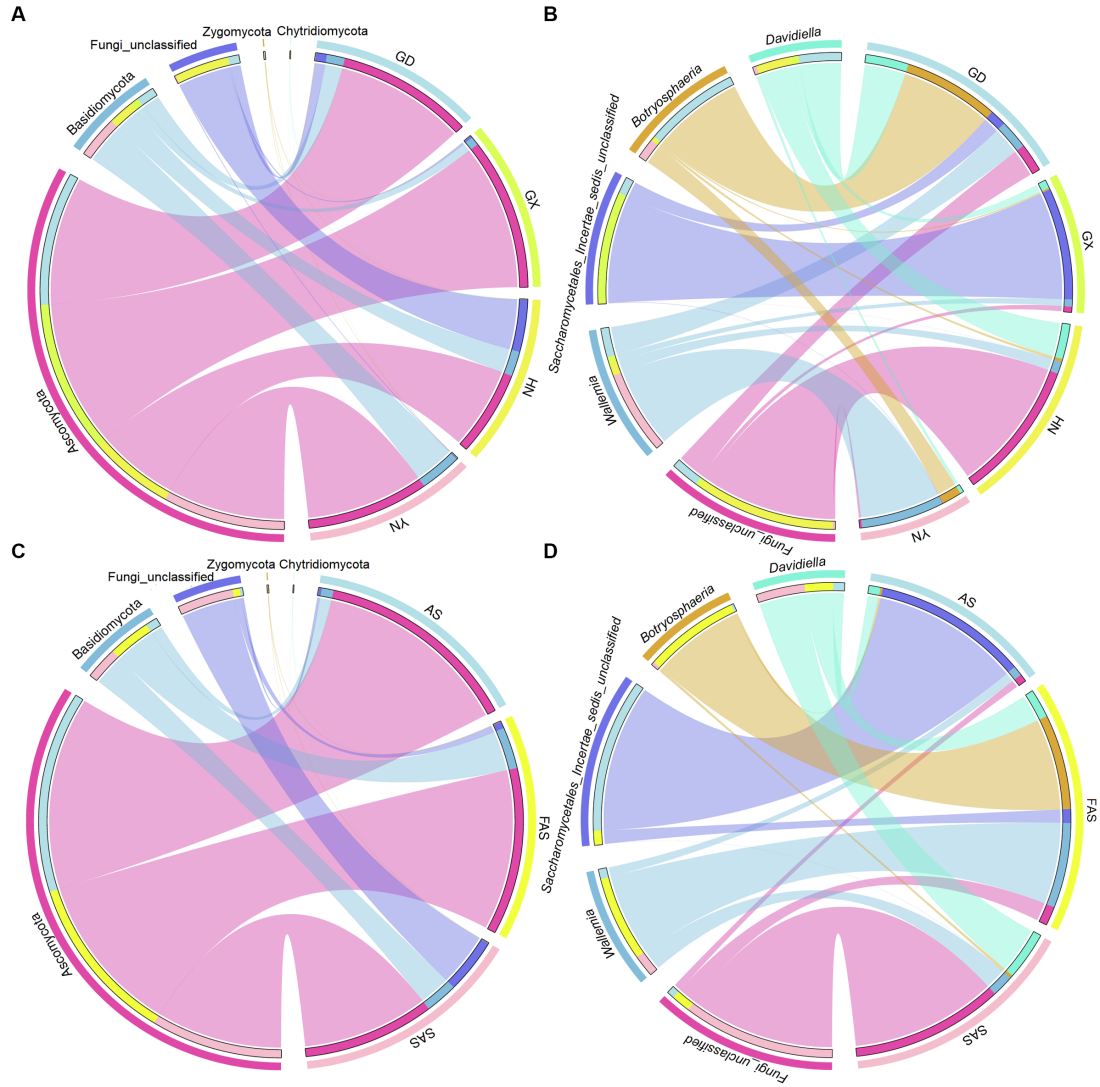
Circos plot fungal community in Arecae semen samples. **(A,B)** Circos plot of predominant taxa at the phylum and genus level based on sampling locations. **(C,D)** Circos plot of predominant taxa at the phylum and genus level based on processing methods.

In accordance with the Venn profiles, 106 shared ASVs were present in four groups on the basis of sampling locations. The unique ASVs numbers in each group were as follows: 485 (GX), 156 (GD), 762 (YN), and 729 (HN) (Supplementary Figure S1A). At the phylum level, Ascomycota (75.45%), Basidiomycota (14.29%), Fungi-unclassified (10.08%), Zygomycota (0.15%), and Chytridiomycota (0.02%) were the top five phyla and presented significant differences in the four areas (*p* < 0.05; Figures 2A, 3A and Supplementary Table S4). Here, Ascomycota had the highest abundance in GX group (93.33% ± 6.59%), followed by GD group (82.32% ± 7.13%) and YN group (73.84% ± 25.86%), and the lowest abundance in HN group (52.32% ± 23.44%). Meanwhile, Basidiomycota had the highest proportion in YN group (25.45% ± 16.02%) and the lowest in HN group (15.40% ± 16.02%). At the genus level, *Fungi_unclassified* (10.08%), *Wallemia* (7.56%), *Saccharomycetales_Incertae_sedis_unclassified* (7.43%), *Botryosphaeria* (6.91%), *Davidiella* (5.14%), *Symbiotaphrina* (4.87%), and *Ceratocystis* (3.87%) were the dominant genera and presented significant region-specific (*p* < 0.05; Figures 2B, 3B and Supplementary Table S4). *Wallemia* were most abundant in YN group (19.18% ± 16.57%), followed by GD group (6.67% ± 4.90%) and HN group (2.74% ± 2.34%), and the lowest in GX group (1.64% ± 1.05%). Meanwhile, *Botryosphaeria* had the highest abundance in GD group (22.02% ± 13.28%) and YN group (4.56% ± 3.53%). In addition, *Davidiella* had highest abundance in GD group (9.86% ± 4.61%) and HN group (8.03% ± 2.29%).

For different processing groups, 789, 485, and 893 ASVs were particularly unique for SAS group, AS group, and FAS group, while the remaining 181 ASVs were shared by three groups (Supplementary Figure S1B). At the phylum level, Ascomycota, Basidiomycota, Fungi-unclassified, Zygomycota, and Chytridiomycota were the top five phyla (Figures 2C, 3C and Supplementary Table S5). The abundance of Ascomycota and Basidiomycota among various processing groups presented significant differences (*p* < 0.05). Ascomycota had higher abundance in the AS group (93.33% ± 6.59%) than in the FAS group (77.42% ± 20.75%) and SAS group (59.58% ± 23.95%). Meanwhile, Basidiomycota had a higher proportion in the FAS group (19.19% ± 5.51%) and SAS group (14.85% ± 9.60%) than in the AS group (5.35% ± 2.99%). The genera *Wallemia*, *Botryosphaeria*, *Davidiella*, *Symbiotaphrina*, and *Ceratocystis* were the dominant genera and exhibited significant differences among three processing groups (*p* < 0.05; Figures 2D, 3D and Supplementary Table S5). *Wallemia*, *Botryosphaeria*, and *Ceratocystis* had higher abundance in the FAS group (14.26% ± 5.50, 15.96% ± 5.03, and 9.18% ± 3.15%, respectively) than in the AS group (1.64% ± 0.68, 0.38% ± 0.11, and 0.20% ± 0.10%, respectively) and SAS group (3.63% ± 0.85, 0.51% ± 0.27, and 0.00% ± 0.00%, respectively). Meanwhile, *Symbiotaphrina* had higher abundance in the AS group (19.39% ± 11.03%) than in the FAS (0.02% ± 0.01%) and SAS (0.06% ± 0.02%) groups.

FUNGuild were conducted to predict the fungal guilds in our samples. The fungal functions could be classified into 24 categories (Supplementary Table S6), of which others accounted for the largest proportion (average 47.44%), followed by Undefined Saprotroph (26.44%), Plant Saprotroph (9.49%), Endophyte (5.29%), Wood Saprotroph (4.79%), Animal Pathogen (2.16%), Dung Saprotroph (1.59%), and Soil Saprotroph (1.11%). The abundances of various guilds varied in our Arecae semen samples samples.

### 3.3. Microbial biomarkers of Arecae semen surface microbiome were shaped by sampling locations and processing methods

**Figure 4:**
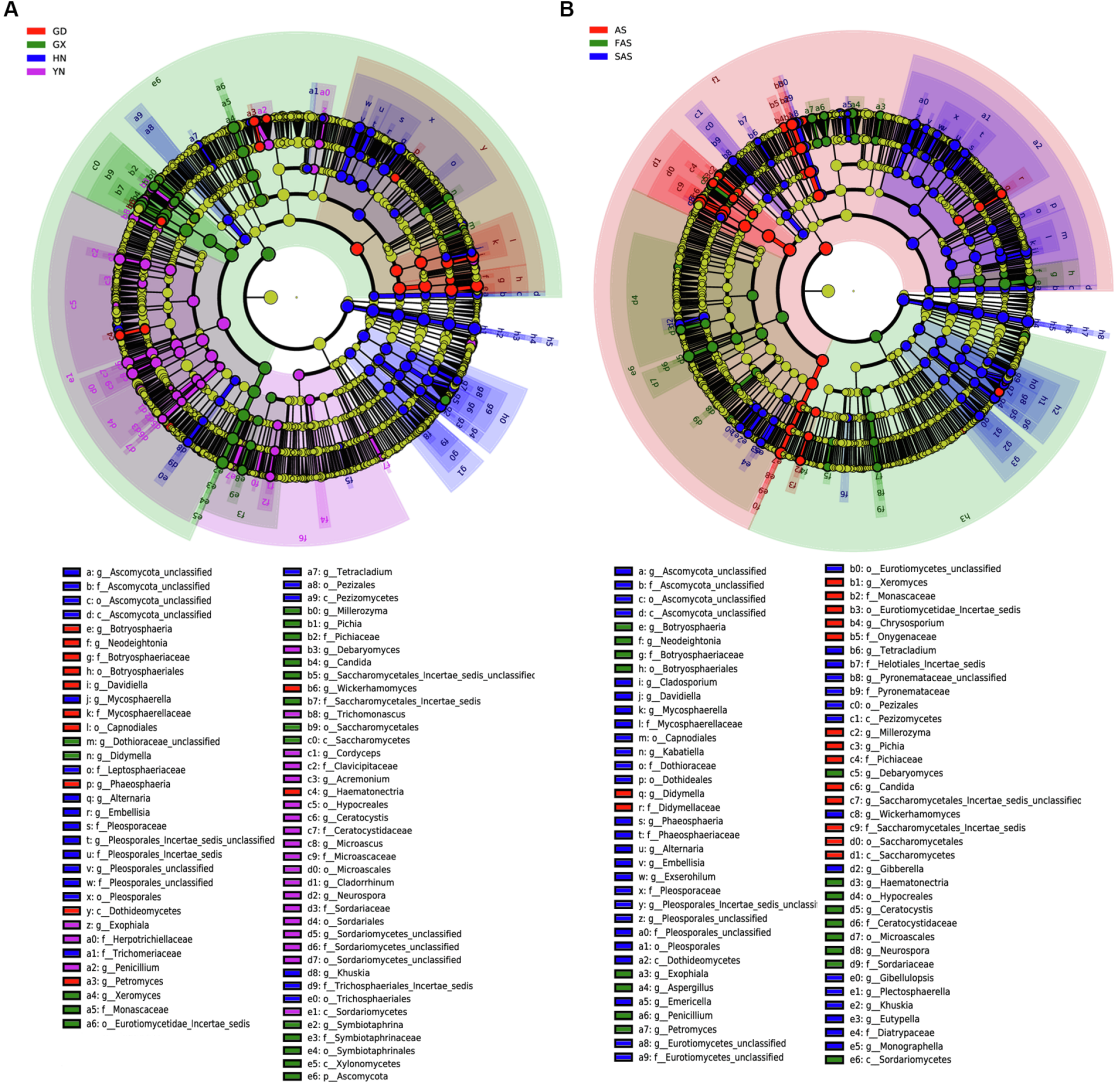
Linear discriminant effect size (LEfSe) of fungal community with a linear discriminant analysis (LDA) score higher than 3.0 and *p*-values less than 0.05 in Arecae semen samples. **(A)** LEfSe based on sampling locations. **(B)** LEfSe based on based on processing methods.

LEfSe revealed differences in community composition among the four sampling locations (Figure 4). Of the 162 bacterial biomarkers (LDA > 3), 19, 34, 60, and 49 were enriched in GD group, GX group, HN group, and AH group, respectively (Figure 4A and Supplementary Figure S2A). The orders Botryosphaeriales and Capnodiales, the class Dothideomycetes, the families Botryosphaeriacetes and Mycosphaerellaceae, the genera *Botryosphaeria*, *Petromyces*, and *Davidiella*, and the species *Lasiodiplodia parva* were enriched in GD group. Meanwhile, the phylum Ascomycota, the orders Saccharomycetales and Symbiotaphrinales, the classes Saccharomycetes and Xylonomycetes, the family Symbiotaphrinaceae, the genera *Symbiotaphina* and *Candida*, and the species *Saccharomycetales incertae sedis*, *Candida tropicalis*, and *Symbiotaphrina kochii* were enriched in GX group. The orders Pleosporales and Tremellales, the classes Tremellomycetes and Microbotryomycetes, the families Pleosporaceae and Tremellaceae, the genera *Alternaria* and *Cryptococcus*, and the species *Alternaria alternata* and *Aureobasidium pullulans* were enriched in HN group. The class Sordariomycetes, the orders Microascales, Hypocreales, and Sordariales, the families Ceratocystidaceae and Microascaceae, the genera *Ceratocystis* and *Microascus*, and the species *Ceratocystis paradoxa* and *Microascus trigonosporus* were enriched in YH group.

LEfSe was also used to reveal potential biomarkers among the three processing groups from phylum to species (Figure 4B and Supplementary Figure S2B). Among the 160 biomarkers (LDA > 3.0), 36, 38, and 86 were enriched in the AS, FAS, and SAS groups, respectively. The phylum Ascomycota, the orders Saccharomycetales, Symbiotaphrinales and Saccharomycetales incertae sedis, the class Xylonomycetes, the families Symbiotaphrinaceae and Monascaceae, the genera *Symbiotaphrina*, *Xeromyces* and *Guehomyces*, and the species *Symbiotaphrina kochii*, *Xeromyces bisporus* and *Candida tropicalis* were enriched in the AS group. Meanwhile, the phylum Basidiomycota, the orders Botryosphaeriales and Microascales, the classes Sordariomycetes and Saccharomycetales, the families Botryosphaeriaceae and Ceratocystidaceae, the genera *Botryosphaeria*, *Ceratocystis* and *Aspergillus*, and the species *Lasiodiplodia parva*, *Wallemia ichthyophaga* and *Ceratocystis paradoxa* were enriched in the FAS group. The orders Pleosporales, Tremellales and Capnodiales, the classes Dothideomycetes and Tremellomycetes, the families Pleosporaceae, Tremellaceae and Mycosphaerellaceae, the genera *Alternaria*, *Davidiella* and *Cryptococcus*, and the species *Alternaria alternata* and *Monascus kaoliang* were enriched in the FAS group.

### 3.4. Network of Arecae semen surface microbiome was shaped by sampling locations and processing methods

**Figure 5:**
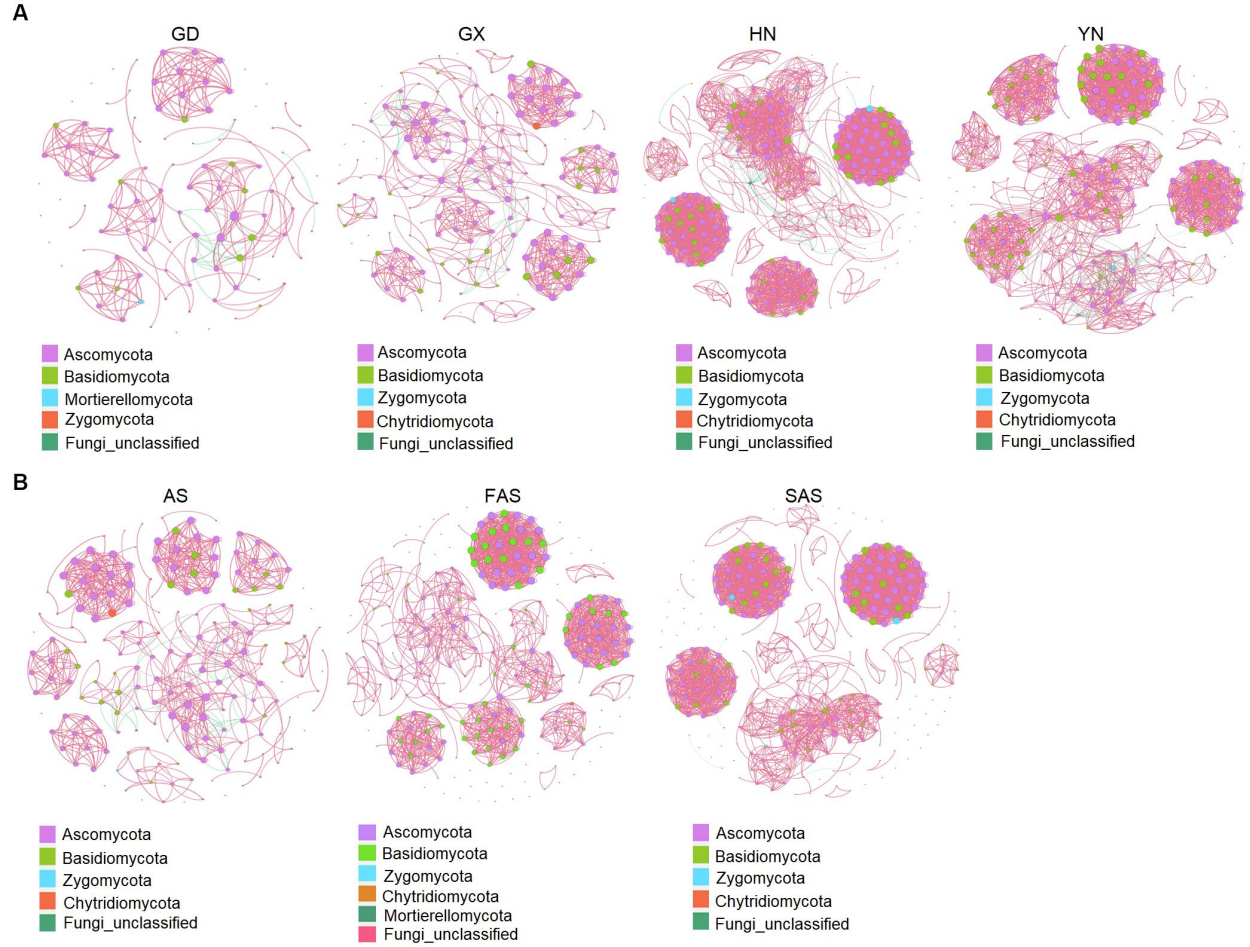
Co-occurrence network analysis of fungal microbial communities in Arecae semen samples (Spearman’s |r| > 0.8 and *p* < 0.05). **(A)** Network based on sampling locations. **(B)** Network based on processing methods. Red and green represent positive and negative correlations, respectively.

The co-occurrence patterns of the fungi communities from the four origins exhibited varying network complexity (as indicated by average degree) and connectivity (Figure 5A and Supplementary Table S7). The average degree was higher in the HN and YH groups (22.46 and 19.96, respectively) than that in the GD and GX groups (6.96 and 8.97, respectively). The values of topological properties (i.e., number of nodes, number of edges, positive edges, number of communities, average degree, average clustering coefficient, average weighted degree, density, and total triangles) were highest in the HN group (300, 3,369, 3,327, 38, 22.46, 0.88, 38.68, 0.08, and 31,706, respectively). Meanwhile, the values of topological properties (i.e., modularity, network diameter, average path length, and modularity with resolution) were highest in the AH group (0.90, 10, 3.56, and 0.90, respectively).

Network complexity (as indicated by average degree) and connectivity were shaped by the processing methods (Figure 5B and Supplementary Table S8). The values of topological properties (i.e., number of nodes, number of edges, positive edges, number of communities, average degree, average clustering coefficient, average weighted degree, density, and total triangles) were highest in the SAS group (308, 2,821, 2,818, 75, 18.32, 0.93, 33.99, 0.06, and 26,726, respectively). The values of topological properties (i.e., negative edges, and modularity and modularity with resolution) were highest in the AS group (40, 0.89, and 0.89, respectively).

## 4. Discussion

The presence of fungi in medicinal herbs has been widely reported, eliciting considerable public concern (Han et al., 2012; Su et al., 2018). For example, the fungal and mycotoxin contamination of 48 medicinal herb samples were investigated and 83.3% of the samples were found to be contaminated with fungi (Chen et al., 2020). The combination of HTS with ITS2 can compensate for the limitations of the culture-based approach and quickly and conveniently analyze the microbial community structure; this technique is frequently used in fungal community analysis to discover unknown and essential microorganisms (Lv et al., 2015; Nilsson et al., 2019a). In previous studies, seed and fructus herbs (i.e., Ziziphi Spinosae semen, Platycladi semen, and Myristicae semen) were found to be more susceptible to fungal contamination as a result of their abundant nutrients (such as carbohydrate nutrients) (Guo et al., 2018; Jiang et al., 2020; Yu et al., 2020). In the current study, HTS and ITS2 were first performed to investigate fungal contamination on the surface of Arecae semen samples. Meanwhile, we compared the differences of the fungal communities in the four groups based on collection origins and the three groups based on processing methods.

Our results showed that Ascomycota and Basidiomycota were the dominant phyla, and *Wallemia*, *Botryosphaeria*, *Davidiella*, *Symbiotaphrina* and *Ceratocystis* were the dominant genera in the Arecae semen samples. The presence of these predominant taxa in other herbs has also been reported. For example, Guo et al. investigated fungal contamination in 14 Ziziphi Spinosae semen samples and found that one phylum (Ascomycota) and three genera (*Aspergillus*, *Candida*, and *Wallemia*) were the most predominant fungi. Those findings are consistent with the results of our study (Guo et al., 2018). Fungal contamination in Lycii Fructus was investigated via ITS2, and the results showed that Ascomycota, Dothideomycetes, Pleosporales, and Pleosporaceae predominated at various levels ranging from family to phylum (Yu et al., 2022a). In the Cassiae semen samples, Ascomycota was the prevailing fungus at the phylum level, and *Aspergillus*, *Cladosporium* and *Penicillium* were the most dominant genera (Guo et al., 2020). Some of the taxa are found to be the dominant genus in herbal medicines for the first time, and the differences between the findings of the present study and previous work may contribute to the distinct nutrient composition of different medicinal substances. *Aspergillus*, *Penicillium*, *Fusarium*, and *Alternaria* are the most common contaminants, and they contain various mycotoxin producers (Ahmad et al., 2014). The existence of potential mycotoxin-producing fungi is an essential condition for mycotoxin contamination. Therefore, the early detection of mycotoxin-producing fungi in medicinal materials is of high significance to prevent further mycotoxin contamination. In our previous study, 323 fungal strains were isolated from herbal medicines, and analysis of potential mycotoxin-producing fungi showed that *Aspergillus flavus* can produce aflatoxins, and *Aspergillus ochraceus* and *Aspergillus niger* can produce ochratoxin A (Wei et al., 2023). Previous reports showed that Arecae semen samples were detected and were positive for aflatoxins and zearalenone (Liu et al., 2016; Liang et al., 2018). So, the fungi should be isolated from the surface of Arecae semen samples in the future, and their ability to produce mycotoxins should be investigated.

The results showed that the diversity, structure, and network of the Arecae semen samples were variable in the four sampling origins in our study. Fungal alpha diversities (Chao 1 and Shannon) across 11 medicinal herb *Platycladus orientalis* samples collected from three provinces (Shandong Province, Anhui Province, and Hebei Province) were compared and presented significant differences (Yu et al., 2020). Fritillariae Cirrhosae Bulbus samples were divided into five groups on the basis of collection areas, and their alpha diversities (Chao 1 and Shannon) exhibited significant difference (Yu et al., 2022b). Similarly, the composition of surface microbiome in our Arecae semen samples collected from four provinces also demonstrated significant differences. Ascomycota had the highest abundance in Guangxi Province, Basidiomycota and *Wallemia* highest in Yunnan Province, while *Botryosphaeria* and *Davidiella* highest in Guangdong Province. Compared with those in the *Platycladus orientalis* samples from Hebei and Shandong provinces, greater unidentified phyla diversity was observed in the samples from Anhui Province, as well as lower Ascomycota diversity (Yu et al., 2020). Differences in the fungal community of the Fritillariae Cirrhosae Bulbus samples were also observed in the five provinces at various taxonomic levels (Yu et al., 2022b). The distribution of these taxa may be related to the local storage conditions in distinct provinces, which may be more suitable for the growth of some taxa than the other sample locations. Different factors (i.e., temperature, humidity, substrate, pH, nutrient availability) in distinct environments may affect the growth of specific fungi (Darko et al., 2018). Such as, *Alternaria* are tolerant to low temperature and usually grow during low temperature transport and storage (EFSA Panel, 2011). High humidity can cause fungi *Fusarium* growth and induces severe mycotoxin contamination (Liao et al., 2020). At low water availability, *Xeromyces* could favor the growth of species in *Aspergillus* and *Eurotium* (Stevenson et al., 2017). In addition, network analyses were performed to explore the interaction patterns of microorganisms in our Arecae semen samples collected from four provinces. We found that positive links among genera were predominant in our network, and network complexity and connectivity were higher in Hainan and Yunnan provinces. These results suggest the potential for extensive cooperative interactions among most taxa in their micro-environments (Qian et al., 2019).

Furthermore, we analyzed the composition of fungal microbiomes on the surface of Arecae semen samples on the basis of processing methods, and differences in fungal communities were observed. The Shannon index was higher in SAS and FAS groups than in AS group. The Chao 1 index in raw Arecae semen was significantly higher than that in the processed samples; this finding is inconsistent with the results of a previous study (Guo et al., 2020). Moreover, Basidiomycota, *Wallemia*, *Botryosphaeria*, and *Ceratocystis* had higher proportion in the FAS and SAS groups than in the AS group. This finding suggests that this fungal microbiome is significantly increased after processing. Moreover, the complexity and connectivity of the network were higher in the processed Arecae semen samples than in the raw samples. This result suggests that the processed Arecae semen samples have a more stable co-occurrence pattern than the raw samples. The reason may be as follows: processing can change the substrate composition of herbs, contribute to the attraction of abundant microorganisms and influence the composition of fungal community, which makes the co-occurrence network of the processed samples more stable (Zheng et al., 2017).

## 5. Conclusion

In this study, the fungal diversity, composition, and network of Arecae semen samples were surveyed using HTS. Here, the diversity and composition of Arecae semen surface microbiome were shaped by sampling location and processing methods. Two phyla (Ascomycota and Basidiomycota), and four genera (*Wallemia*, *Botryosphaeria*, *Davidiella*, and *Symbiotaphrina*) were the dominant fungus, and they presented significant differences in the four areas and the three processed products. The α-diversity and network complexity exhibited differences in the four sampling locations and three processed samples. This study highlights the importance of the roles of sampling locations and processing methods in Arecae semen surface microbiome.

## Data availability statement

The datasets presented in this study can be found in online repositories. The names of the repository/repositories and accession number(s) can be found in the article/Supplementary material.

## Author contributions

GW analyzed the data and drafted the manuscript. JX performed the wet-lab experiments. ZZ collected the samples. GZ analyzed the data and revised the manuscript critically. LD and SC coordinated the study, granted funds, and participated in the drafting and revision of the manuscript. All authors contributed to the article and approved the submitted version.

## Funding

This study was supported by grants from the National Key R&D Plan (2022YFC3501801, 2022YFC3501804 and No. 2018YFC1706302), Fundamental Research Funds for the Central public welfare research institutes (No. ZXKT21037, No. ZZ15-YQ-044, No. ZXKT22050, and No. ZXKT22001), Scientific Research Project of Hainan Academician Innovation Platform (SQ2021PTZ0052).

## Conflict of interest

The authors declare that the research was conducted in the absence of any commercial or financial relationships that could be construed as a potential conflict of interest.

## Publisher’s note

All claims expressed in this article are solely those of the authors and do not necessarily represent those of their affiliated organizations, or those of the publisher, the editors and the reviewers. Any product that may be evaluated in this article, or claim that may be made by its manufacturer, is not guaranteed or endorsed by the publisher.
