# Supplementary material for: Sampling locations and processing methods shape fungi microbiome on the surface of edible and medicinal Arecae semen
Source: Front Microbiol. 2023 Jul 20;14:1188986. doi: 10.3389/fmicb.2023.1188986 (PMC10397402; doi:10.3389/fmicb.2023.1188986)
Supplement: Supplementary file 1 [file Data_Sheet_1.doc]

**Fig. S1** Venn diagram of fungal community in Arecae semen samples. (A) Venn diagram based on sampling locations. (B) Venn diagram based on processing methods.

**
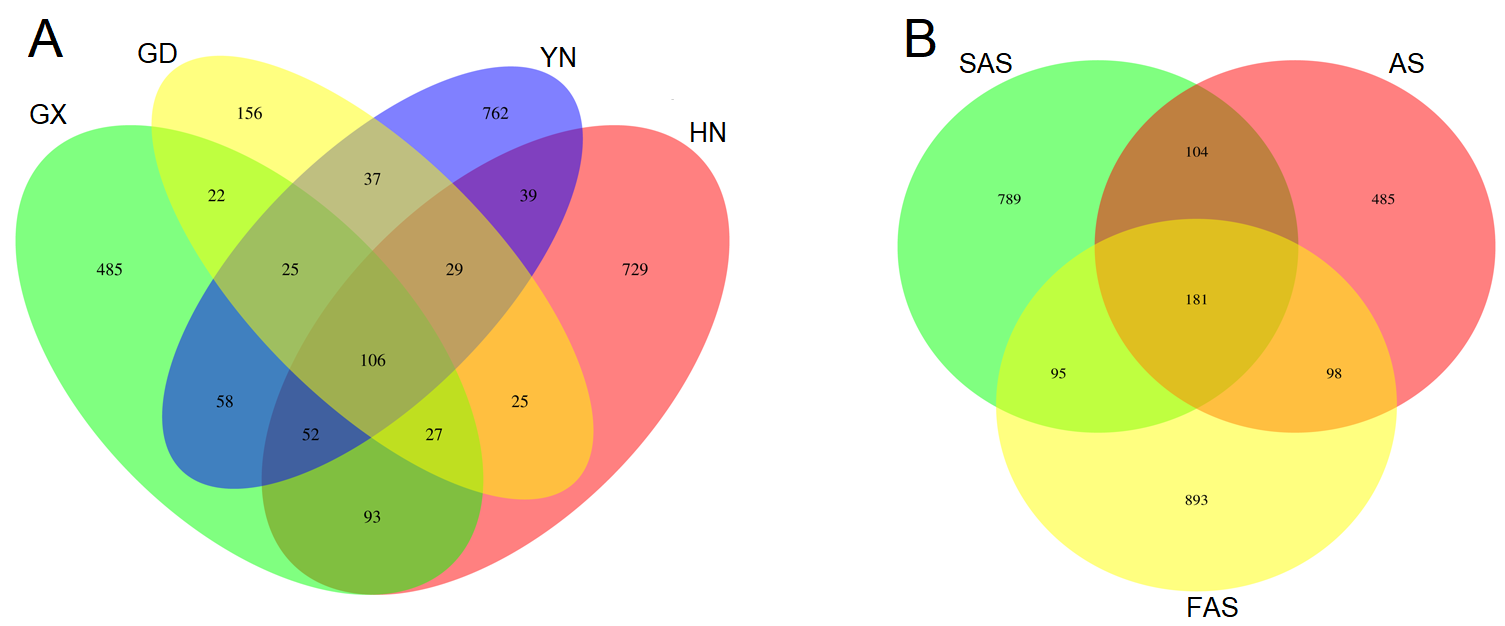
**

**Fig. S2** The linear discriminant analysis (LDA) score higher than 3.0 and *P* values less than 0.05 of fungal community in Arecae semen samples based on sampling locations (A) and processing methods (B).

**
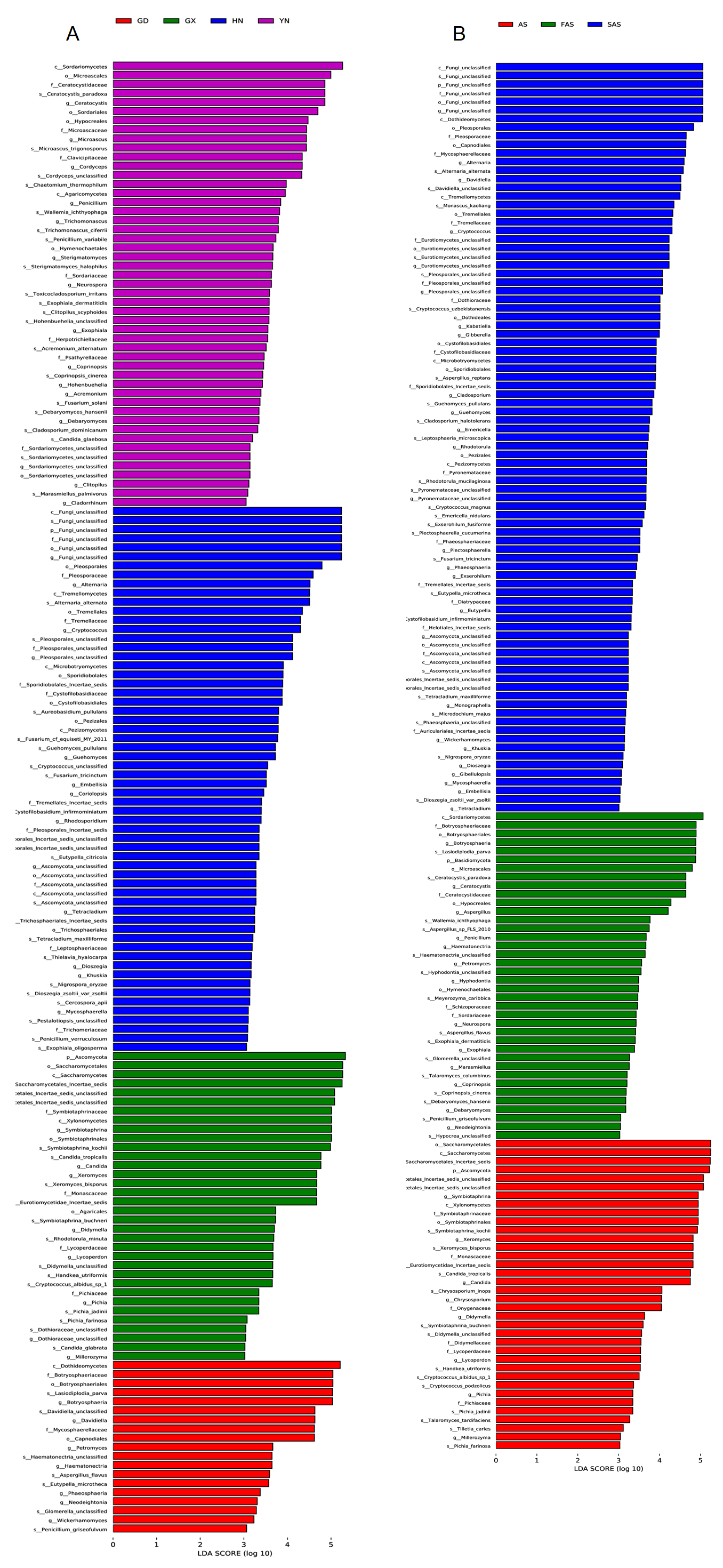
**

**Table S1** Voucher information of crude and slices of Arecae semen samples

| Voucher number | Sources | Sampling location | Processing |
| --- | --- | --- | --- |
| 1_1 | Baise,  Guangxi Province | GX | Raw Arecae Semen (AS) |
| 1_2 | Baise,  Guangxi Province | GX | Raw Arecae Semen (AS) |
| 1_3 | Baise,  Guangxi Province | GX | Raw Arecae Semen (AS) |
| 2_1 | Haikou,  Hainan Province | HN | Arecae Semen Tostum (SAS) |
| 2_2 | Haikou,  Hainan Province | HN | Arecae Semen Tostum (SAS) |
| 2_3 | Haikou,  Hainan Province | HN | Arecae Semen Tostum (SAS) |
| 3_1 | Nanning,  Guangxi Province | GX | Raw Arecae Semen (AS) |
| 3_2 | Nanning,  Guangxi Province | GX | Raw Arecae Semen (AS) |
| 3_3 | Nanning,  Guangxi Province | GX | Raw Arecae Semen (AS) |
| 4_1 | Guangzhou,  Guangdong Province | GD | Arecae Semen Carbonisata (FAS) |
| 4_2 | Guangzhou,  Guangdong Province | GD | Arecae Semen Carbonisata (FAS) |
| 4_3 | Guangzhou,  Guangdong Province | GD | Arecae Semen Carbonisata (FAS) |
| 5_1 | Sanya,  Hainan Province | HN | Arecae Semen Tostum (SAS) |
| 5_2 | Sanya,  Hainan Province | HN | Arecae Semen Tostum (SAS) |
| 5_3 | Sanya,  Hainan Province | HN | Arecae Semen Tostum (SAS) |
| 6_1 | Kunming,  Yunnan Province | YN | Arecae Semen Carbonisata (FAS) |
| 6_2 | Kunming,  Yunnan Province | YN | Arecae Semen Carbonisata (FAS) |
| 6_3 | Kunming,  Yunnan Province | YN | Arecae Semen Carbonisata (FAS) |
| 7_1 | Yuxi,  Yunnan Province | YN | Arecae Semen Carbonisata (FAS) |
| 7_2 | Yuxi,  Yunnan Province | YN | Arecae Semen Carbonisata (FAS) |
| 7_3 | Yuxi,  Yunnan Province | YN | Arecae Semen Carbonisata (FAS) |
| 8_1 | Shenzhen, Guangdong Province | GD | Arecae Semen Carbonisata (FAS) |
| 8_2 | Shenzhen, Guangdong Province | GD | Arecae Semen Carbonisata (FAS) |
| 8_3 | Shenzhen, Guangdong Province | GD | Arecae Semen Carbonisata (FAS) |
| 9_1 | Qionghai,  Hainan Province | HN | Arecae Semen Tostum (SAS) |
| 9_2 | Qionghai,  Hainan Province | HN | Arecae Semen Tostum (SAS) |
| 9_3 | Qionghai,  Hainan Province | HN | Arecae Semen Tostum (SAS) |
| 10_1 | Foshan,  Guangdong Province | GD | Arecae Semen Tostum (SAS) |
| 10_2 | Foshan,  Guangdong Province | GD | Arecae Semen Tostum (SAS) |
| 10_3 | Foshan,  Guangdong Province | GD | Arecae Semen Tostum (SAS) |
| 11_1 | Liuzhou,  Guangxi Province | GX | Raw Arecae Semen (AS) |
| 11_2 | Liuzhou,  Guangxi Province | GX | Raw Arecae Semen (AS) |
| 11_3 | Liuzhou,  Guangxi Province | GX | Raw Arecae Semen (AS) |
| 12_1 | Lijiang,  Yunnan Province | YN | Arecae Semen Carbonisata (FAS) |
| 12_2 | Lijiang,  Yunnan Province | YN | Arecae Semen Carbonisata (FAS) |
| 12_3 | Lijiang,  Yunnan Province | YN | Arecae Semen Carbonisata (FAS) |

**Table S2** The α-diversity in Arecae semen samples based on origin.

| Indices | GX | Significance | HN | Significance | GD | Significance | YN | Significance | F | *P* |
| --- | --- | --- | --- | --- | --- | --- | --- | --- | --- | --- |
| Chao 1 | 166.11±18.94 | ab | 198.27±44.91 | a | 79.08±14.05 | b | 213.45±41.84 | a | 3.35 | 0.03 |
| Shannon | 3.64±0.48 | b | 4.61±0.0.61 | a | 3.57±0.34 | b | 4.21±0.30 | ab | 1.208 | 0.04 |

**Table S3** The α-diversity in Arecae semen samples based on processing.

| Indices | AS | Significance | SAS | Significance | FAS | Significance | F | *P* |
| --- | --- | --- | --- | --- | --- | --- | --- | --- |
| Chao 1 | 166.11±18.65 | a | 167.80±36.85 | a | 160.23±31.12 | a | 0.017 | 0.983 |
| Shannon | 3.64±0.48 | a | 4.54±0.47 | a | 3.80±0.26 | a | 1.452 | 0.249 |

**Table S4** The most abundant fungal communities in Arecae semen samples based on sampling location.

|  | Microbiome | GD | Significance | GX | Significance | HN | Significance | YN | Significance | F | *P* |
| --- | --- | --- | --- | --- | --- | --- | --- | --- | --- | --- | --- |
| Phylum | Ascomycota | 82.32±7.13 | a | 93.33±6.59 | a | 52.33±23.44 | b | 73.84±25.86 | a | 8.259 | 0.000 |
|  | Basidiomycota | 10.94±4.40 | ab | 5.35±2.99 | b | 15.40±10.93 | ab | 25.45±16.02 | a | 3.056 | 0.042 |
|  | Fungi_unclassified | 6.51±3.79 | b | 1.25±0.21 | b | 32.07±30.77 | a | 0.50±0.11 | b | 7.914 | 0.000 |
|  | Zygomycota | 0.22±0.11 | a | 0.05±0.04 | a | 0.13±0.08 | a | 0.21±0.18 | a | 0.893 | 0.455 |
|  | Chytridiomycota | 0.00±0.00 | a | 0.01±0.01 | a | 0.06±0.03 | a | 0.03±0.022 | a | 0.857 | 0.474 |
| Genus | Fungi_unclassified | 6.51±3.79 | b | 1.24±1.20 | b | 32.07±20.76 | a | 0.51±0.34 | b | 7.914 | 0.000 |
|  | Wallemia | 6.67±4.9 | ab | 1.64±1.05 | b | 2.74±2.34 | ab | 19.18±16.57 | a | 3.151 | 0.038 |
|  | Saccharomycetales_Incertae_sedis_unclassified | 3.80±2.19 | b | 25.35±20.35 | a | 0.02±0.02 | b | 0.28±0.11 | b | 5.376 | 0.004 |
|  | Botryosphaeria | 22.02±13.28 | a | 0.37±0.32 | b | 0.67±0.51 | b | 4.56±3.53 | b | 6.601 | 0.001 |
|  | Davidiella | 9.86±4.61 | a | 1.87±0.86 | b | 8.03±2.29 | a | 0.79±0.45 | b | 2.915 | 0.045 |

**Table S5** The most abundant fungal communities in Arecae semen samples based on processing methods.

|  | Microbiome | AS | Significance | FAS | Significance | SAS | Significance | F | *P* |
| --- | --- | --- | --- | --- | --- | --- | --- | --- | --- |
| Phylum | Ascomycota | 93.33±6.59 | a | 77.42±20.75 | ab | 59.58±23.95 | b | 7.749 | 0.002 |
|  | Basidiomycota | 5.35±2.99 | b | 19.19±5.51 | a | 14.85±9.60 | a | 2.325 | 0.014 |
|  | Fungi_unclassified | 1.25±0.40 | b | 3.16±1.73 | b | 25.36±8.35 | a | 7.074 | 0.003 |
|  | Zygomycota | 0.06±0.01 | a | 0.21±0.08 | a | 0.15±0.05 | a | 1.222 | 0.308 |
|  | Chytridiomycota | 0.01±0.01 | a | 0.00±0.00 | a | 0.05±0.03 | a | 0.794 | 0.461 |
| Genus | Fungi_unclassified | 1.25±0.40 | b | 3.16±1.73 | b | 25.36±8.35 | a | 7.074 | 0.003 |
|  | Wallemia | 1.64±0.68 | b | 14.26±5.50 | a | 3.63±0.85 | b | 2.983 | 0.044 |
|  | Saccharomycetales_Incertae_sedis_unclassified | 25.75±10.45 | a | 2.35±1.30 | b | 0.02±0.00 | b | 8.174 | 0.001 |
|  | Botryosphaeria | 0.38±0.11 | b | 15.96±5.03 | a | 0.51±0.27 | b | 6.514 | 0.004 |
|  | Davidiella | 1.87±0.86 | a | 4.76±2.98 | a | 8.07±1.74 | a | 1.428 | 0.258 |
|  | Symbiotaphrina | 19.39±11.03 | a | 0.02±0.01 | b | 0.06±0.02 | b | 4.760 | 0.015 |
|  | Ceratocystis | 0.20±0.10 | b | 9.18±3.15 | a | 0.00±0.00 | b | 5.723 | 0.007 |

**Table S6 Fungal guilds were predicted among various samples using FUNGuild.**

| Samples | Algal Parasite | Animal Endosymbiont | Animal Pathogen | Bryophyte Parasite | Dung Saprotroph | Ectomycorrhizal | Endophyte | Endosymbiont | Epiphyte | Ericoid Mycorrhizal | Fungal Parasite | Insect Pathogen | Leaf Saprotroph | Lichen Parasite | Lichenized | Litter Saprotroph | Nematophagous | Plant Pathogen | Plant Saprotroph | Soil Saprotroph | Undefined Parasite | Undefined Saprotroph | Wood Saprotroph | other |
| --- | --- | --- | --- | --- | --- | --- | --- | --- | --- | --- | --- | --- | --- | --- | --- | --- | --- | --- | --- | --- | --- | --- | --- | --- |
| AC1_1 | 0.00 | 0.15 | 1.60 | 0.00 | 0.21 | 0.01 | 1.65 | 0.21 | 0.21 | 0.00 | 0.60 | 0.00 | 0.00 | 0.22 | 0.00 | 0.25 | 0.00 | 3.87 | 0.00 | 0.24 | 0.00 | 22.57 | 0.57 | 67.66 |
| AC1_2 | 0.00 | 0.00 | 1.69 | 0.00 | 0.00 | 0.01 | 0.74 | 0.06 | 0.06 | 0.00 | 0.49 | 0.00 | 0.00 | 0.14 | 0.00 | 0.44 | 0.00 | 1.78 | 0.00 | 0.06 | 0.00 | 11.13 | 0.65 | 82.74 |
| AC1_3 | 0.00 | 0.00 | 0.11 | 0.00 | 0.01 | 0.00 | 0.10 | 0.01 | 0.01 | 0.00 | 0.01 | 0.00 | 0.00 | 0.01 | 0.00 | 0.00 | 0.00 | 0.30 | 0.01 | 0.02 | 0.01 | 1.82 | 0.06 | 97.51 |
| AC3_1 | 0.00 | 0.01 | 4.03 | 0.00 | 4.49 | 0.00 | 7.96 | 3.50 | 3.50 | 0.00 | 0.19 | 0.00 | 0.00 | 0.03 | 0.03 | 0.00 | 0.00 | 0.82 | 0.00 | 7.93 | 0.00 | 52.13 | 0.18 | 15.19 |
| AC3_2 | 0.00 | 0.00 | 10.39 | 0.00 | 0.70 | 0.00 | 17.81 | 10.03 | 10.03 | 0.00 | 0.02 | 0.00 | 0.00 | 0.01 | 0.00 | 0.00 | 0.00 | 0.35 | 0.00 | 10.71 | 0.00 | 33.16 | 0.20 | 6.60 |
| AC3_3 | 0.00 | 0.01 | 4.43 | 0.00 | 2.96 | 0.01 | 11.31 | 4.00 | 4.00 | 0.00 | 0.18 | 0.00 | 0.00 | 0.01 | 0.00 | 0.00 | 0.00 | 0.43 | 0.00 | 6.91 | 0.00 | 45.16 | 0.13 | 20.46 |
| AC11_1 | 0.00 | 0.39 | 1.41 | 0.00 | 0.70 | 0.05 | 1.46 | 0.05 | 0.05 | 0.00 | 0.26 | 0.01 | 0.00 | 0.00 | 0.00 | 0.03 | 0.00 | 2.34 | 0.00 | 0.25 | 0.00 | 11.29 | 0.78 | 80.95 |
| AC11_2 | 0.00 | 0.39 | 2.14 | 0.00 | 0.11 | 0.00 | 1.26 | 0.04 | 0.04 | 0.00 | 0.32 | 0.00 | 0.00 | 0.00 | 0.00 | 0.00 | 0.00 | 3.27 | 0.00 | 0.07 | 0.00 | 11.89 | 0.54 | 79.94 |
| AC11_3 | 0.00 | 0.34 | 2.25 | 0.00 | 0.43 | 0.10 | 7.31 | 0.06 | 0.06 | 0.00 | 0.46 | 0.00 | 0.00 | 0.00 | 0.00 | 0.00 | 0.00 | 2.91 | 0.10 | 0.11 | 0.00 | 17.04 | 1.39 | 67.42 |
| AC4_1 | 0.00 | 0.00 | 6.10 | 0.00 | 0.35 | 0.01 | 22.56 | 0.00 | 0.00 | 0.00 | 0.00 | 0.00 | 0.00 | 0.00 | 0.00 | 0.00 | 0.00 | 21.94 | 0.00 | 0.01 | 0.00 | 8.63 | 22.29 | 18.12 |
| AC4_2 | 0.00 | 0.00 | 3.86 | 0.00 | 0.26 | 0.00 | 13.99 | 0.00 | 0.00 | 0.00 | 0.00 | 0.00 | 0.00 | 0.00 | 0.00 | 0.00 | 0.00 | 13.72 | 0.00 | 0.11 | 0.00 | 12.41 | 12.56 | 43.08 |
| AC4_3 | 0.00 | 0.00 | 0.72 | 0.00 | 2.30 | 0.00 | 7.55 | 0.02 | 0.02 | 0.00 | 0.04 | 0.00 | 0.00 | 0.00 | 0.00 | 0.00 | 0.00 | 2.29 | 0.04 | 2.21 | 0.00 | 26.17 | 1.91 | 56.72 |
| AC6_1 | 0.00 | 0.00 | 3.47 | 0.00 | 0.78 | 0.00 | 2.23 | 0.20 | 0.20 | 0.00 | 1.79 | 0.00 | 0.00 | 0.00 | 0.26 | 0.00 | 0.00 | 17.02 | 0.19 | 0.25 | 0.00 | 24.85 | 6.90 | 41.88 |
| AC6_2 | 0.00 | 0.00 | 6.63 | 0.00 | 0.43 | 0.00 | 8.21 | 0.00 | 0.00 | 0.00 | 0.00 | 0.00 | 0.00 | 0.00 | 0.00 | 0.00 | 0.00 | 27.43 | 0.00 | 0.00 | 0.00 | 10.22 | 27.71 | 19.36 |
| AC6_3 | 0.00 | 0.00 | 2.27 | 0.00 | 3.26 | 0.01 | 3.88 | 0.26 | 0.26 | 0.00 | 0.00 | 0.00 | 0.00 | 0.00 | 0.00 | 0.00 | 0.00 | 10.81 | 0.00 | 0.26 | 0.00 | 29.29 | 7.82 | 41.88 |
| AC7_1 | 0.00 | 0.02 | 0.67 | 0.00 | 0.28 | 0.00 | 1.28 | 0.01 | 0.01 | 0.00 | 0.10 | 0.00 | 0.016 | 0.01 | 0.03 | 0.02 | 0.00 | 14.20 | 0.10 | 0.02 | 0.00 | 67.99 | 9.38 | 5.87 |
| AC7_2 | 0.00 | 0.00 | 0.39 | 0.00 | 0.45 | 0.02 | 1.51 | 0.01 | 0.01 | 0.00 | 0.03 | 0.00 | 0.01 | 0.04 | 0.11 | 0.00 | 0.00 | 16.65 | 0.25 | 0.21 | 0.00 | 62.27 | 11.77 | 6.27 |
| AC7_3 | 0.00 | 0.00 | 2.78 | 0.00 | 1.27 | 0.00 | 1.83 | 0.00 | 0.01 | 0.00 | 0.00 | 0.00 | 0.00 | 0.00 | 0.00 | 0.00 | 0.00 | 18.52 | 1.17 | 0.00 | 0.00 | 51.92 | 16.01 | 6.48 |
| AC8_1 | 0.00 | 0.00 | 0.00 | 0.00 | 0.23 | 0.00 | 18.20 | 0.00 | 0.00 | 0.00 | 0.00 | 0.00 | 0.00 | 0.00 | 0.00 | 0.00 | 0.00 | 25.49 | 0.00 | 0.00 | 0.00 | 20.07 | 16.37 | 19.63 |
| AC8_2 | 0.00 | 0.00 | 0.00 | 0.00 | 0.18 | 0.00 | 9.08 | 0.00 | 0.00 | 0.00 | 0.00 | 0.00 | 0.00 | 0.00 | 0.00 | 0.00 | 0.00 | 16.25 | 0.00 | 0.00 | 0.00 | 63.28 | 8.52 | 2.69 |
| AC8_3 | 0.00 | 0.00 | 0.00 | 0.00 | 0.05 | 0.00 | 8.05 | 0.00 | 0.00 | 0.00 | 0.00 | 0.00 | 0.00 | 0.00 | 0.00 | 0.00 | 0.00 | 22.89 | 0.00 | 0.00 | 0.00 | 20.10 | 7.95 | 40.95 |
| AC12_1 | 0.001 | 0.00 | 0.06 | 0.00 | 1.17 | 0.01 | 1.09 | 0.01 | 0.01 | 0.00 | 0.02 | 0.00 | 0.00 | 0.00 | 0.00 | 0.00 | 0.00 | 4.51 | 0.13 | 0.08 | 0.00 | 1.66 | 3.35 | 87.93 |
| AC12_2 | 0.002 | 0.00 | 0.11 | 0.00 | 23.24 | 0.00 | 2.34 | 0.00 | 0.03 | 0.00 | 0.06 | 0.00 | 0.00 | 0.01 | 0.00 | 0.01 | 0.00 | 4.43 | 0.04 | 0.15 | 0.00 | 4.33 | 2.41 | 62.83 |
| AC12_3 | 0.004 | 0.00 | 0.14 | 0.00 | 2.58 | 0.00 | 1.70 | 0.00 | 0.00 | 0.00 | 0.05 | 0.00 | 0.00 | 0.00 | 0.01 | 0.00 | 0.00 | 5.17 | 0.09 | 0.19 | 0.00 | 3.87 | 3.37 | 82.80 |
| AC2_1 | 0.000 | 0.00 | 3.94 | 0.00 | 0.55 | 0.00 | 5.74 | 0.04 | 0.06 | 0.00 | 0.23 | 0.00 | 0.04 | 0.06 | 1.66 | 0.18 | 0.00 | 16.36 | 0.03 | 0.39 | 0.00 | 35.89 | 1.04 | 33.80 |
| AC2_2 | 0.017 | 0.08 | 2.92 | 0.02 | 0.68 | 0.00 | 5.10 | 0.07 | 0.08 | 0.00 | 0.21 | 0.00 | 0.00 | 0.19 | 0 | 0.05 | 0.00 | 14.42 | 0.16 | 0.25 | 0.00 | 35.34 | 2.29 | 38.14 |
| AC2_3 | 0.00 | 0.04 | 4.30 | 0.00 | 0.33 | 0.00 | 3.71 | 0.04 | 0.11 | 0.00 | 0.91 | 0.00 | 0.00 | 0.09 | 0.22 | 0.01 | 0.00 | 14.43 | 0.10 | 0.14 | 0.00 | 32.71 | 1.36 | 41.50 |
| AC5_1 | 0.00 | 0.00 | 0.18 | 0.00 | 1.44 | 0.00 | 1.50 | 0.00 | 0.01 | 0.23 | 0.08 | 0.00 | 0.00 | 0.00 | 0.00 | 0.08 | 0.00 | 0.96 | 0.00 | 1.44 | 0.00 | 11.49 | 0.27 | 82.32 |
| AC5_2 | 0.00 | 0.00 | 0.02 | 0.00 | 1.25 | 0.00 | 2.08 | 0.01 | 0.01 | 0.00 | 0.01 | 0.00 | 0.00 | 0.00 | 0.00 | 0.01 | 0.00 | 0.70 | 0.00 | 1.28 | 0.00 | 10.22 | 0.10 | 84.30 |
| AC5_3 | 0.00 | 0.00 | 0.26 | 0.00 | 0.96 | 0.00 | 0.89 | 0.00 | 0.00 | 0.00 | 0.02 | 0.00 | 0.00 | 0.00 | 0.00 | 0.02 | 0.00 | 0.74 | 0.00 | 0.96 | 0.00 | 8.68 | 0.22 | 87.25 |
| AC9_1 | 0.00 | 0.00 | 0.88 | 0.00 | 0.02 | 0.01 | 2.89 | 0.00 | 0.00 | 0.00 | 0.00 | 0.00 | 0.00 | 0.00 | 0.00 | 0.00 | 0.00 | 10.62 | 0.00 | 0.00 | 0.00 | 22.56 | 2.73 | 60.28 |
| AC9_2 | 0.00 | 1.37 | 4.84 | 0.00 | 0.42 | 0.00 | 2.16 | 0.00 | 1.93 | 0.00 | 0.25 | 0.00 | 0.00 | 0.00 | 0.00 | 0.00 | 0.00 | 7.97 | 0.25 | 0.25 | 0.00 | 19.76 | 1.19 | 59.59 |
| AC9_3 | 0.00 | 0.79 | 2.43 | 0.00 | 0.02 | 0.00 | 1.69 | 0.01 | 0.01 | 0.00 | 0.53 | 0.00 | 0.00 | 0.00 | 0.00 | 0.53 | 0.00 | 11.03 | 0.00 | 0.01 | 0.00 | 23.91 | 0.03 | 59.00 |
| AC10_1 | 0.00 | 0.00 | 0.00 | 0.00 | 1.63 | 0.00 | 1.52 | 0.00 | 0.00 | 0.00 | 0.00 | 0.00 | 0.00 | 0.00 | 0.00 | 0.00 | 0.00 | 3.62 | 0.00 | 1.63 | 0.00 | 55.21 | 0.00 | 36.38 |
| AC10_2 | 0.00 | 0.17 | 2.17 | 0.00 | 2.80 | 0.00 | 3.71 | 0.33 | 0.33 | 0.00 | 1.27 | 0.00 | 0.00 | 0.00 | 0.28 | 0.00 | 0.00 | 5.09 | 0.00 | 3.10 | 0.00 | 42.48 | 0.13 | 38.14 |
| AC10_3 | 0.00 | 0.49 | 0.49 | 0.00 | 0.84 | 0.00 | 6.40 | 0.00 | 0.00 | 0.00 | 0.00 | 0.00 | 0.00 | 0.00 | 0.00 | 0.00 | 0.00 | 18.48 | 0.00 | 0.69 | 0.00 | 40.32 | 0.15 | 32.12 |
| Average | 0.00 | 0.12 | 2.16 | 0.00 | 1.59 | 0.01 | 5.29 | 0.53 | 0.58 | 0.01 | 0.23 | 0.00 | 0.00 | 0.02 | 0.07 | 0.05 | 0.00 | 9.49 | 0.07 | 1.11 | 0.00 | 26.44 | 4.79 | 47.44 |

**Table S7** Topological properties of co-occurring fungal networks within Arecae semen samples based on sampling location.

| Network properties | GD | GX | HN | YN |
| --- | --- | --- | --- | --- |
| Number of nodes | 106 | 189 | 300 | 257 |
| Number of edges | 369 | 848 | 3369 | 2308 |
| Positive edges | 352 | 810 | 3327 | 2253 |
| Negative edges | 17 | 38 | 42 | 55 |
| Modularity | 0.84 | 0.90 | 0.76 | 0.82 |
| Number of communities | 31 | 31 | 38 | 31 |
| Network diameter | 9 | 10 | 9 | 8 |
| Average path length | 3.14 | 3.56 | 2.95 | 3.20 |
| Average degree | 6.96 | 8.97 | 22.46 | 17.96 |
| Average clustering coefficient | 0.86 | 0.85 | 0.88 | 0.87 |
| Average weighted degree | 9.33 | 12.80 | 38.68 | 28.95 |
| Density | 0.07 | 0.05 | 0.08 | 0.07 |
| Modularity with resolution | 0.84 | 0.90 | 0.76 | 0.82 |
| Total triangles | 625 | 2054 | 31706 | 14234 |

**Table S8** Topological properties of co-occurring fungal networks within Arecae semen samples based on processing methods.

| Network properties | AS | FAS | SAS |
| --- | --- | --- | --- |
| Number of nodes | 188 | 270 | 308 |
| Number of edges | 867 | 1791 | 2821 |
| Positive edges | 827 | 1790 | 2818 |
| Negative edges | 40 | 1 | 3 |
| Modularity | 0.89 | 0.80 | 0.74 |
| Number of communities | 32 | 63 | 75 |
| Network diameter | 10 | 10 | 8 |
| Average path length | 3.56 | 3.68 | 2.04 |
| Average degree | 9.22 | 13.27 | 18.32 |
| Average clustering coefficient | 0.85 | 0.90 | 0.93 |
| Average weighted degree | 13.17 | 23.78 | 33.99 |
| Density | 0.050 | 0.050 | 0.06 |
| Modularity with resolution | 0.89 | 0.80 | 0.74 |
| Total triangles | 2192 | 9997 | 26726 |
